# Vinegar in Opium Poisoning

**Published:** 1877-11

**Authors:** J. D. Fawcett

**Affiliations:** Baltimore, Md.


### VINEGAR IN OPIUM POISONING.

From the *Medical Brief.*

In the July number of the *Medical Brief*, I notice an article, headed *Poisoning by Morphia*, reported by Dr. Chapman, of a gentleman having taken morphia by mistake, etc.

I do not wish to criticise the Doctor's treatment, but will give my experience in two cases: one, having taken ten grains of morphia, and the other, about forty grains of opium. I simply give my treatment for its simplicity and hasty relief in restoring and counteracting the above-mentioned drugs.

Case first—Was called to see a lady who had taken forty grains of opium. I arrived at the bedside in half an hour after the dose was taken; found her in a stupor; I immediately called for some strong vinegar; succeeded in getting 6 ounces into her stomach; then bathed her all over with the same, rubbing it in with my hand. In half an hour I had her completely restored.

Case second—We called to see a gentleman having taken ten grains of morphia, for the purpose of committing suicide. I reached the case in thirty minutes after the morphia had been taken; gave the patient two wineglasses of strong vinegar at intervals of five minutes aparts, and he was restored in thirty minutes. This mode of treatment is very simple, and the specific remedy is always at hand. Respectfully,

Baltimore, Md. J. D. FAWCETT, M. D.

